# Environmental conditions for Jamestown Canyon virus correlated with population-level resource selection by white-tailed deer in a suburban landscape

**DOI:** 10.1371/journal.pone.0223582

**Published:** 2019-10-07

**Authors:** Karmen M. Hollis-Etter, Robert A. Montgomery, Dwayne R. Etter, Christopher L. Anchor, James E. Chelsvig, Richard E. Warner, Paul R. Grimstad, Diane D. Lovin, Marvin S. Godsey

**Affiliations:** 1 Biology Department, University of Michigan-Flint, Flint, Michigan, United States of America; 2 Department of Fisheries and Wildlife, Michigan State University, East Lansing, Michigan, United States of America; 3 Wildlife Division Michigan Department of Natural Resources, Lansing, Michigan, United States of America; 4 Forest Preserve District of Cook County, River Forest, Illinois, United States of America; 5 Natural Resources and Environmental Sciences, University of Illinois Champaign-Urbana, Urbana, Illinois, United States of America; 6 Department of Biological Sciences, University of Notre Dame, Notre Dame, Indiana, United States of America; 7 Division of Vector-Borne Diseases, Centers for Disease Control and Prevention, Fort Collins, Colorado, United States of America; Tufts University Cummings School of Veterinary Medicine, UNITED STATES

## Abstract

Suburban landscapes can alter spatial patterns by white-tailed deer (*Odocoileus virginianus*) and increase animal contact with vectors, pathogens, and humans. Close-contact relationships at a landscape level can have broad implications for disease epidemiology. From 1995–1999, we captured and radio-collared 41 deer in two suburban forest preserves in Chicago, Illinois. We collected blood to determine if animals were seronegative or seropositive for Jamestown Canyon virus and tracked deer movements within suburban habitats. We developed utilization distributions at the population-level and evaluated resource selection for seronegative and seropositive deer. We used maximum likelihood estimation for model selection via Akaike information criterion and then restricted maximum likelihood estimation to attain unbiased estimates of the parameters in the top-ranking models. The top-ranking model describing the resource selection of seronegative deer received almost the full weight of evidence (Akaike information criterion *ω*_*i*_ = 0.93), and included the proportion of wetlands, precipitation in year *t*, and an interaction of the proportion of wetlands and precipitation in year *t*. The top-ranking model describing resource selection of seropositive deer received the full weight of evidence (Akaike information criterion *ω*_*i*_ = 1.00). The model included distance to nearest populated place, distance to nearest river, length of road in each grid cell, precipitation in year *t*, and an interaction of the length of road in each grid cell and precipitation in year *t*. These results are valuable for mapping the spatial configuration of hotspots for Jamestown Canyon virus and could be used to educate local residents and recreationalists to reduce human exposure.

## Introduction

Most emerging human diseases are zoonotic and involve animal hosts and/or vectors at some stage of transmission [1]. The rising number of emerging zoonoses may be driven by modernized farming practices especially in the developing world, habitat destruction, human encroachment, and climate change [2]. Urbanization can also lead to the emergence of zoonotic diseases and increase risk factors for disease transmission to humans [3–4]. Ecological factors can precipitate disease emergence and transmission by placing people in contact with a natural reservoir or host by increased proximity [5–6].

Urban systems are a network of interfaces in which pathogens can be transmitted between animals and humans [3]. Wildlife in urban environments have the potential to persist at high densities and act as a reservoir for a broad array of disease-causing pathogens [7]. Many zoonotic studies focusing on surveillance of seropositive animals fail to consider the ecology of the host wildlife species in the host-vector-pathogen relationship. Habitat use of different wildlife species can vary both temporally and spatially [8–10] so it would be expected that vector-pathogen exposure would vary among species. Understanding how a host species uses available habitat under varying ecological conditions could assist managers in predicting disease spread and persistence [11]. This is particularly important in urban landscapes where the potential for human exposures to pathogens is increased.

Jamestown Canyon Virus (JCV) is classified as a zoonotic disease [12] and humans are susceptible to infection when co-occupying landscapes where favorable environmental conditions support vector and host wildlife species. Approximately 105 arboviral diseases and 300 mosquito vector species are associated with arbovirus transmission [13]. In Connecticut, human seroprevalence for JCV ranges from 3.9% to 10.1% [14]. Patriquin et al [15] estimated 20.6% of the human population in Nova Scotia, Canada were infected with JCV and human seropositivity reached 48.2% in one region. White-tailed deer (*Odocoileus virginianus*) are recognized as the principal amplification host and the virus has been isolated from at least 22 species of mosquitoes and *Aedes* and *Ochlerotatus* spp appear to be the primary vectors for JCV, depending on geography [16].

Temporal and spatial overlap between hosts and vectors are key requirements for the emergence of transmitted vector-borne pathogens and identifying spatially explicit environmental conditions that lead to potential disease risks for humans. We examined landscape level resource selection by suburban white-tailed deer as a means of evaluating the spatial distribution of JCV in two forest preserves in Chicago, Illinois. Our objective was to determine if spatially explicit hotspots for JCV can be identified by examining how a host (deer) utilizes the landscape.

## Methods and materials

### Study areas

**Fig 1:**
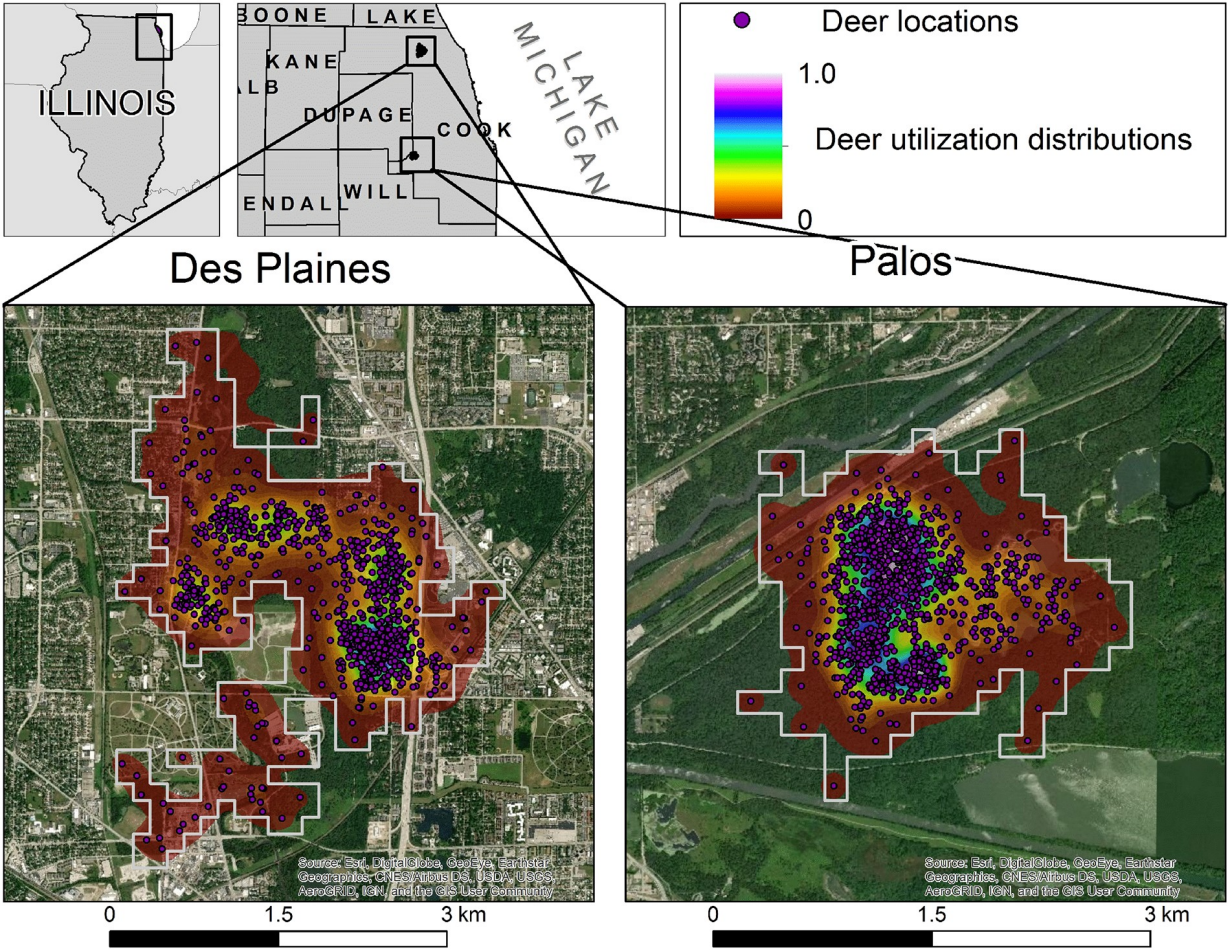
Distribution of the study areas for assessing the resource selection of deer in Cook County, Illinois (1996–1999).

We evaluated the prevalence of JCV and evaluated population-level resource selection of seronegative and seropositive white-tailed deer radio-collared from 1996–1999 in two suburban forest preserves in Cook County, Illinois (Fig 1). These study areas are part of the 27,499 ha Forest Preserve District of Cook County (FPDCC) located in suburban Chicago, Illinois (41°85' N, 87°65' W). The forest preserves act as ecological islands where deer persist amidst various extremes from wooded, industrial development, or heavily suburbanized environments.

They contain over 200 picnic areas, 161 km of bike trails, lakes, rivers, and 323 km of multiuse trails. The northerly Des Plaines (DP) site is a 781 ha forest preserve along the Des Plaines River in northwestern Cook County containing 48% developed land, 44% forest, and 2% wetlands [17]. The southerly Palos site is a 435 ha forest preserve in southwestern Cook County which occurs at the fork of the Des Plaines River and the Chicago Sanitary and Calumet Shipping Canals. Palos primary land cover categories include 72% forest, 11% wetlands, 10% grasslands but only 5.4% of the land is developed. The two study sites were separated by approximately 41 linear kilometers.

This project was part of a broader deer population ecology study in urban forest preserves in Chicago, Illinois. These two study sites were originally chosen because they were of comparable size, had large populations of urban deer, and deer management was ongoing in both preserves. The two sites provided an interesting comparison in that the amount of anthropogenic development varied between the forest preserves [18].

Land cover within the two study sites contained various water sources and wetlands providing potential unique breeding habitats for disease vector mosquitoes. These included rivers, low-lying pools adjacent to rivers, slow moving streams, lakes, floodplains, and prairie potholes which may hold water for extended periods of time, depending on snowmelt or rainfall. Both study sites also contained wooded uplands with mature trees which can provide water-retaining tree holes for larval habitats for many pathogen vectors, especially mosquitoes [19].

Regional climate is temperate, consisting of warm, humid summers and cold winters. The average high daily temperature is 28°C during the midsummer months and -10.4°C as the low in January. Mean annual rainfall is 84.9 cm and annual snowfall is 97.3 cm [20].

### Deer capture

We captured deer with drop-nets (Wildlife Materials Inc., Carbondale, Illinois) [21] and remote dart gun (Pneu-Dart Inc., Williamsport, Pennsylvania) [22] from December to March (1995–1998). Netted deer were anesthetized with xylazine hydrochloride (2.0 mg/kg Cervazine^®^, Wildlife Pharmaceuticals Inc., Fort Collins, Colorado) and darted deer with tiletamine/zolazepam hydrochloride (4.4 mg/kg Telazol^®^, Fort Dodge Laboratories, Fort Dodge, Iowa) and 2.0 mg/kg xylazine hydrochloride. Xylazine hydrochloride was antagonized with yohimbine hydrochloride (0.25 mg/kg Antagonil^®^, Wildlife Pharmaceuticals Inc., Fort Collins, Colorado) [23]. All live-captured deer were marked with two numbered plastic ear tags for visual identification and with metal ear tags with FPDCC return information. Selected female deer were fitted with radio-collars equipped with an 8-hour, time delayed, mortality switch (Advanced Telemetry Systems, Isanti, Minnesota; Telonics, Mesa, Arizona).

Physiological information (i.e., age and sex) was recorded from all captured deer. Deer age was determined by tooth replacement and wear as fawn (<1 year old), yearling (1–2 years old), or adult (≥2 years old) [24]. Deer capture locations were recorded on topographic images and later transferred to ArcGIS software (Environmental Systems Research Institute Inc., Redlands, California) to create a geographic information system (GIS). Blood was collected (10–20 ml) and sera was stored at -60°C.

This study was carried out in accordance with the guidelines by the University of Illinois Laboratory Animal Care Advisory Committee under the protocol approvals # V5R246 and V5R246/8340. Animal capture and handling procedures established by the American Veterinary Medical Association and American Society of Mammalogists were followed. Anesthesia was applied as described in the methods. All ecological permits were obtained from the Illinois Department of Natural Resources and Forest Preserve District of Cook County.

### Radio-telemetry

All radio-collared females were radio-tracked to calculate home ranges and examine movement patterns. We located deer 1–3 times per week via triangulation using two truck mounted, four-element yagi antennas, aligned in a null configuration [25]. Triangulations were entered into Locate II^®^ software 1.3 (Pacer, Truro, Nova Scotia, Canada) [26]. To determine deer locations, the maximum likelihood estimator was used from ≥ 3 radio-bearings collected within a 20 minute interval. The 90^th^ percentile of deer location error ellipses (11.5 ha) was used as the upper limit for acceptable error and locations with larger error were deleted [27].

### Serology

For detection of La Crosse (LAC) and JC virus antibodies, sera were initially tested by CAL group virus IgG captured enzyme linked immunosorbent assays (ELISA). Samples were determined as positive for CAL group antibodies (LAC or JC) when the mean absorbance for the three test wells was ≥ 2 times the mean of the negative control. If positive for more than one CAL group virus, samples were retested using neutralization assays. Titers were compared to differentiate closely related viruses. The higher antibody titer was used to indicate virus specificity when > 4 fold difference in antibody titer was detected against closely related viruses [28–29]. We coded the deer with a binary covariate representing individual deer condition (COND) with deer that were seronegative for JCV (COND = 0) and deer that were seropositive (COND = 1).

### Deer resource selection

Our objective was to evaluate third-order [30] resource selection of white-tailed deer. We divided our telemetry relocation efforts into three temporal periods each spanning 12 months from May through April. This temporal period coincides with the life cycle of mosquitos and deer, and facilitated analysis of the precipitation data in year *t* and *t-1*. In Illinois, deer home-range area and movements are reduced in the summer months [25–27] and mosquito abundance was reported to peak in July in Kansas [31]. To correspond with the life cycles of both deer and mosquitoes, we radio-tracked deer from May through April 1996–1997, 1997–1998, and 1998–1999. To define the spatial extent of deer space use within the forest preserves we developed utilization distributions (UDs) based on the spread of deer telemetry locations within the study areas. We calculated the UDs using a bivariate plug-in matrix allowing for parameter smoothing along rotated axes [32–34]. We then clipped a grid lattice at a 150 m^2^ cell resolution to the spatial extent of the UD within each forest preserve to ensure that modeling efforts only considered habitat within the population range of the deer (Fig 1). From our sample of telemetered deer, we used deer locations to identify grid cells used by deer. A used/available design [35–36] was most appropriate for our data given that the number of deer tracked annually varied throughout the duration of our study (Table 1) [37–39]. For example, a continuous response variable, representing number of deer locations in each cell mapping intensity of use [40–43], would have been inappropriate because of the variation in sampling of individual deer. We calculated the UDs as fixed-kernel density estimates using a bivariate plug-in matrix allowing for parameter smoothing along rotated axes [40–42]. We considered grid cells to be used when at least one deer was observed during the year of study. Correspondingly, available grid cells were those were no deer locations were detected in the specific year of study. We made these calculations for seropositive and seronegative deer separately and this binary used/available metric functioned as the response variable in our spatially explicit mixed linear regression model.

**Table 1:** The number of deer radio-tracked, number of deer per site (DP = Des Plaines), and number of telemetry locations for deer seronegative or seropositive for Jamestown Canyon virus in Cook County, Illinois (1996–1999).

	Seronegative		Seropositive	
Year	No. of deer tracked (per site)	No. of telemetry locations	No. of deer tracked (per site)	No. of telemetry locations
**1996–1997**	16 (10 DP, 6 Palos)	307	6 (4 DP, 2 Palos)	82
**1997–1998**	24 (8 DP, 16 Palos)	421	14 (10 DP, 4 Palos)	220
**1998–1999**	15 (5 DP, 10 Palos)	530	10 (8 DP, 2 Palos)	364

### Environmental conditions

To estimate resource selection of deer, we described the landscape structure of the forest preserves by collating information on a suite of environmental conditions at the same resolution of the grid lattice (i.e., 150 m^2^). Using a raster based land cover layer with a native resolution of 30 m x 30 m [17, 44–45] we calculated the amount of edge (EDGE) in each grid cell in our study areas. We classified edge habitat according to the adjoining habitat types which could influence the habitat selection of deer. Thus, we represented EDGE by the transition from *upland* habitat type (primarily deciduous canopy) to *partial canopy/savannah* (more open areas). Next, we considered stagnant wetlands (STAG), because STAG can serve as breeding grounds for mosquitoes [46–47]. Based on the distribution of wetlands delineated by the National Wetlands Inventory [48], we calculated the proportion of each grid cell that was covered by floodplain forest, wet meadow, shallow and deep marshes, and any surface waters not connected to a flowing river. We merged and dissolved polygons representing each of these stagnant water features, intersected the resultant polygon with the 150 m^2^ grid lattice, and calculated the proportion of each cell that was covered in wetlands. To test for the effect of roads, we calculated the length (in meters) of roads within each grid cell (ROAD).

To test the proximity of deer space use to specific environmental conditions we calculated nearest distance from the center of each grid cell to various environmental features. In this capacity we calculated distance to rivers (RIVER) to test whether space use was greater for seronegative deer in the vicinity of moving water. We also evaluated the effect of anthropogenic disturbance by calculating distance to the boundary (BOUND) of each forest preserve and distance to populated places (POP) or permanent human development as defined by the United States Geological Survey (USGS) [49]. We interpreted the boundary to be the edge dividing green space (i.e., the forest reserves) and impervious surfaces emblematic of the surrounding built environment. We then calculated the nearest distance to the built environment from each grid cell. The USGS layer also features a set of populated places (i.e., villages, towns, or cities) [50] emblematic of permanent human development. Once again we calculated the nearest distance from each grid cell to the populated places.

To understand the spatial variability in precipitation levels we downloaded precipitation data from five weather stations distributed in the vicinity of our study areas [51]. We calculated cumulative precipitation levels associated with the temporal period of our data collection efforts (i.e., May to April of each year of study). Our division of the year was consistent across both the deer location data and the precipitation data. To capture the cumulative weather station data at the level of each grid cell, we absorbed the precipitation (PRECIP) data from the nearest weather station. To do so, we conducted a join based on distance. It is important to note that we did not have a cell that was precisely equidistant between two weather stations. Anticipating that there might be a lag effect associated with the saturation of habitat with water and mosquito breeding, we considered PRECIP in year *t-1*, as well as in time *t*. Finally, there were two interaction terms that we hypothesized could affect the resource selection of deer that were positive for JCV. We hypothesized that habitat with more precipitation and a higher proportion of wetlands would be correlated with the resource selection of deer that were seropositive for JCV (STAG * PRECIP). Conversely, we expected the resource selection of seronegative deer to be associated with habitat with less precipitation and a lower proportion of wetlands (i.e., habitat that was drier). Additionally, we hypothesized that habitat with more precipitation and greater road length would be correlated with resource selection of deer that were seropositive for JCV (ROAD * PRECIP). Rain-filled trenches alongside roads and divots made by car tracks can be productive breeding grounds for mosquitoes [52–53]. We hypothesized that seropositive deer resource selection would be associated with habitat containing greater road length and more precipitation.

### Modeling design

We fit a spatially explicit mixed linear regression model with a logit link which took the form;

$${Logit(Y}_{i})=x_{i}^{\prime }\beta +Z_{i}$$

with *Y*_*i*_ representing the binary response variable (used or available) in each *i*th grid cell, *x*_*i*_ are vectors of the environmental conditions at each *i*th grid cell, β represents vectors of the model parameters while *Z*_*i*_ is the random error term that assumes a spatially autocorrelated structure. We fit this model in SAS (SAS Institute version 9.2, Cary, North Carolina) using PROC GLIMMIX. The model attributed random variation at the forest preserve level and allowed for separate correlative error processes by year of the study (N = 3).

The suite of hypothesized models we developed and tested were based on *a priori* rationale representing environmental correlates for seropositive deer resource selection. The first 14 of these models included main effects only. The remaining six models included a combination of main effects and the two interaction terms. Finally, we were interested in determining whether there was a lag effect associated with precipitation levels. Therefore, any of these existing 20 models where the covariate (PRECIP *t*) was present, we exchanged it with PRECIP *t-1*. This substitution provided an additional 15 models for testing.

To justify evaluating deer resource selection separately by seronegative or seropositive, we developed a global model with COND as a covariate. Documentation of significant interaction terms between COND and the environmental covariates would identify that deer responded to that environmental condition differently by condition and would support running models separately for deer that were seronegative or seropositive for JCV. In this respect parameter significance was set at α < 0.15 level, a common p-value for retaining parameters in model selection capacities [54].

We used maximum likelihood estimation for model selection via Akaike Information Criterion (AIC) and then restricted maximum likelihood estimation to attain unbiased estimates of the parameters in the top-ranking models [55]. To evaluate the effect of the model parameters in the top-ranking model(s), we rescaled logit output from 0 to 1 representing the relative probability of resource selection [56–57].

## Results

We radio-tracked 41 female deer > 6 months-of-age from 1996–1999 collecting a total of 1,924 telemetry locations from DP and Palos. We tracked 22 deer in 1996–1997, 38 deer in 1997–1998, and 25 deer in 1998–1999 (Table 1). Sixty-six percent of radio-tracked deer (N = 27) were seronegative and 34% (N = 14) were seropositive for JCV. We evaluated collinearity and determined that none of our environmental covariates were correlated (r > 0.70). Our initial modeling efforts identified that 4 of 8 environmental covariates significantly (α < 0.15) interacted with viral status (Table 2). These terms demonstrated variation in deer resource selection by JCV status. Therefore, we elected to evaluate the resource selection of seronegative and seropositive deer separately. We standardized all continuous predictors to allow comparison between models and tested the 35 models for seronegative deer and the same 35 models for seropositive deer.

**Table 2:** Parameter estimates and standard errors (SE) for the interaction of Jamestown Canyon virus serostatus and environmental covariates describing suburban forest preserves in Cook County, Illinois (1996–1999). Significant (α = 0.15) interaction terms identify variation in selection of the environmental covariate by serostatus (seronegative or seropositive for Jamestown Canyon virus).

Effect	Main effect	*P*	Condition effect	*P*	Interaction effect	*P*
BOUND * COND	0.17 (0.07)	< .001	0.52 (0.08)	< .001	0.26 (0.08)	< .001
STAG * COND	0.10 (0.06)	0.01	0.54 (0.08)	< .001	0.000 (0.08)	0.99
RIVER * COND	0.26 (0.06)	< .001	0.54 (0.08)	< .001	0.04 (0.08)	0.60
EDGE * COND	0.40 (0.06)	< .001	0.57 (0.09)	< .001	-0.09 (0.08)	0.26
ROAD * COND	0.10 (0.06)	0.01	0.54 (0.08)	< .001	0.02 (0.08)	0.77
POP * COND	-0.52 (0.07)	< .001	0.56 (0.09)	< .001	0.64 (0.09)	< .001
PRECIP *t* * COND	-0.26 (0.14)	0.06	0.54 (0.08)	< .001	0.20 (0.09)	0.02
PRECIP *t-1* * COND	0.77 (0.21)	< .001	0.50 (0.09)	< .001	0.13 (0.09)	0.15

BOUND is distance forest preserve boundary, COND is Jamestown Canyon virus serostatus of deer (0 = seronegative, 1 = seropositive), STAG is proportion of wetlands in each grid cell, RIVER is distance to nearest river, EDGE is length of edge habitat in each grid cell, ROAD is length of road in each grid cell, POP is distance to nearest populated place, PRECIP is total precipitation level in year *t* or *t-1*.

**Fig 2:**
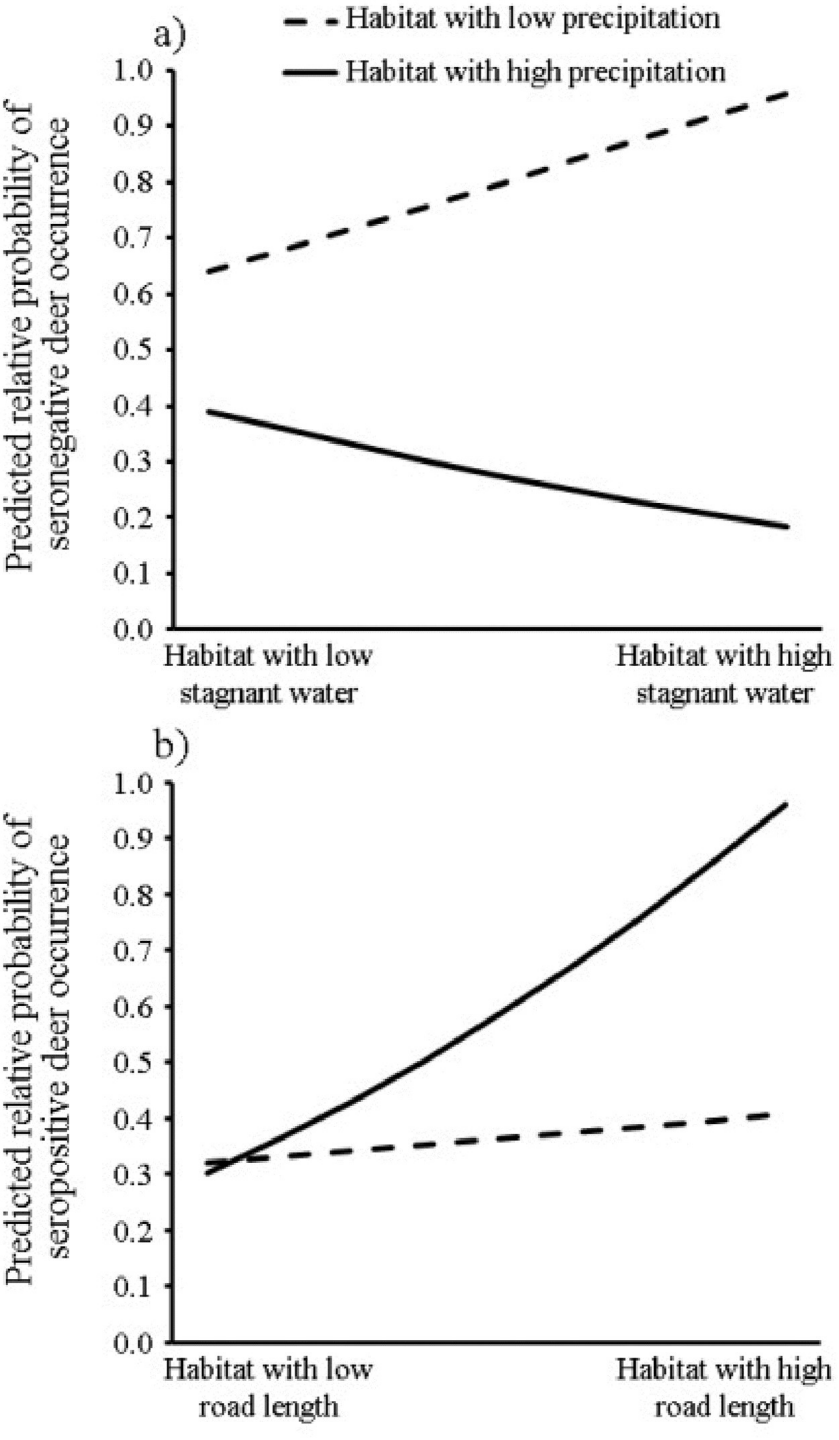
Plots of the magnitude of effects for the interaction terms featured in the top-ranking models describing the resource selection of deer that were seronegative (panel a) and seropositive (panel b) for Jamestown Canyon virus, Cook County, Illinois (1996–1999).

The top-ranking model describing the resource selection of seronegative deer received almost the full weight of evidence (AIC *ω*_*i*_ = 0.93, Table 3). No other model considered had ΔAIC < 2 indicating lack of support. The resource selection of seronegative deer was best described by a model that included the proportion of wetlands (STAG), precipitation in year *t* (PRECIP *t*), and an interaction of the proportion of wetlands and precipitation in year *t* (STAG * PRECIP *t*, Table 3). The relative probability of seronegative deer resource selection was significantly affected by this interaction term (*P* = 7.22 × 10^−3^, Table 4). Specifically, the relative probability of selection for seronegative deer was highest in habitat that had low precipitation levels in year *t* and a greater proportion of wetlands (Fig 2a). The relative probability of selection was lowest in habitat with greater precipitation levels in year *t* and a greater proportion of wetlands (Fig 2a).

**Table 3:** The top five models describing resource selection of deer seronegative and seropositive for Jamestown Canyon virus in Des Plaines and Palos forest preserves of Cook County Illinois, 1996–1999.

	AIC	ΔAIC	ω
**Seronegative models**			
STAG + PRECIP *t* + STAG * PRECIP *t*	6396.17	0.00	0.93
**STAG + POP + ROAD + PRECIP *t***	6401.45	5.28	0.07
**RIVER + STAG + PRECIP *t***	6414.06	17.89	0.00
**STAG + RIVER + POP + ROAD + PRECIP *t***	6419.55	23.38	0.00
POP + RIVER + ROAD + PRECIP *t* + ROAD * PRECIP *t*	6421.05	24.88	0.00
**Seropositive models**			
POP + RIVER + ROAD + PRECIP *t* + ROAD * PRECIP *t*	6911.96	0.00	1.00
**STAG + POP + ROAD + PRECIP *t***	6923.28	11.32	0.00
**STAG + RIVER + POP + ROAD + PRECIP *t***	6932.80	20.84	0.00
STAG + PRECIP *t* + STAG * PRECIP *t*	6954.27	42.31	0.00
**EDGE + ROAD + POP + PRECIP *t***	6958.30	46.34	0.00

BOUND is distance forest preserve boundary, COND is Jamestown Canyon virus serostatus deer (0 = seronegative, 1 = seropositive), STAG is proportion of wetlands in each grid cell, RIVER is distance to nearest river, EDGE is length of edge habitat in each grid cell, ROAD is length of road in each grid cell, POP is distance to nearest populated place, PRECIP is total precipitation level in year *t* or *t-1*.

**Table 4:** Parameter estimates and standard errors (SE) for the environmental covariates featured in the top-ranking models describing the resource selection of deer that were seronegative and seropositive for Jamestown Canyon virus, Cook County, Illinois (1996–1999). Significant parameters (α = 0.05) are in bold type face.

Parameter estimate (SE)							
	POP	RIVER	ROAD	STAG	PRECIP *t*	STAG * PRECIP *t*	ROAD * PRECIP *t*
**Seronegative**	---	---	---	0.09 (0.06)	-0.32 (0.18)	**-0.17 (0.06)**	---
**Seropositive**	**-0.73 (0.10)**	**0.40 (0.07)**	**0.27 (0.07)**	---	0.11 (0.21)	---	**0.12 (0.06)**

POP is distance to nearest populated place, RIVER is distance to nearest river, ROAD is length of road in each grid cell, STAG is proportion of wetlands in each grid cell, PRECIP *t* is total precipitation level in year *t*.

The top-ranking model describing the resource selection of seropositive deer received the full weight of evidence (AIC *ω*_*i*_ = 1.00, Table 3). All remaining models had ΔAIC < 2, indicating lack of support. The resource selection of seropositive deer was best described by a model that included the distance to nearest populated place (POP), the distance to nearest river (RIVER), the length of road in each grid cell (ROAD), precipitation in year *t* (PRECIP *t*), and an interaction of the length of road in each grid cell and precipitation in year *t* (ROAD * PRECIP *t*, Table 3). The relative probability of seropositive deer resource selection was negatively related to the distance to nearest populated place (*P* = 7.53×10^−14^, Table 4) and was positively related to the distance to nearest river (*P* = 1.71×10^−8^, Table 4). Additionally, the relative probability of seropositive deer resource selection was significantly affected by the interaction term (*P* = 0.05, Table 4). Specifically, the relative probability of selection for seropositive deer was highest in habitat that had high precipitation levels in year *t* and a greater road length per grid cell (Fig 2b). The relative probability of selection was lowest in habitat with less road length per grid cell and this effect was virtually identical regardless of habitat precipitation in year *t* (Fig 2b).

## Discussion

Many emerging infectious disease events are caused by vector-borne pathogens of wildlife origins [58] and transmission of these pathogens involves interactions among the vector, host species, and their environment. For example, Johnson et al [59] confirmed that favorable ecological conditions facilitated West Nile Virus (WNV) transmission among mosquito and avian hosts in an urban environment. We examined how a host (deer) interacted with spatially explicit environmental conditions that could lead to potential JCV risk for humans in a suburban environment.

Our results of differing resource selection for seronegative and seropositive deer is supported by the complex female white-tailed deer social structure that influences annual individual deer movements and habitat use [25, 60–61]. Female family groups and sometimes unrelated does associate frequently during fall and winter [25]; however, during parturition (May through July), pregnant females segregate from related and unrelated does and establish fawning sites in preferred habitats. Females that are barren (mostly the previous year’s offspring) have home-ranges that may overlap slightly with their dams, but primarily they co-occupy areas that are not defended by a dominant doe [60–61]. This seasonal segregation and admixing results in different annual individual female deer home-ranges and use of variable habitats [25, 27].

None of the top-10 ranking models for seronegative or seropositive deer featured PRECIP *t-1* indicating that there was no lag-time effect associated with precipitation levels. However, there needs to be more work examining how long after infection JCV titers can be detected in deer. Issel et al [62] determined that titers peaked by day 5 in deer experimentally infected with JCV but remained detectable beyond 180 days.

For seronegative deer, there was an important interaction effect between PRECIP in year *t* and the proportion of wetlands (STAG) per grid cell. Specifically, seronegative deer tended not to select habitat that was high in the proportion of STAG in years when that habitat received high levels of precipitation. This finding is not surprising given that female deer typically avoid wetland habitats during the summer [25, 63–64]. Nixon et al [25] hypothesized that deer avoidance of wetlands in summer could be related to biting insect abundance as deer reduced use of bottom-land forests when biting insect (including mosquitoes) populations were 15 times higher in bottom-land forests than adjacent standing corn. However, the data set of animal locations used to develop the models for seronegative deer was about twice as large in two sampling years as that for seropositive deer, which could influence model performance.

With respect to the interaction term ROAD * PRECIP *t*, seropositive deer selection was highest in habitat that had higher proportion of road length per grid cell in years when the habitat received more precipitation. Puddles along roads have been identified as primary breeding habitat for mosquitos [52–53] and during the summer deer frequent roadsides for grazing [65–66] and fawning cover [63] increasing the chance to contact vectors and pathogen. Our study results would suggest higher prevalence of JCV in suburban areas with increased road densities. The finding of 34% prevalence is comparable to rural populations from Wisconsin [67] and Indiana [28], however annual fluctuations in prevalence of JCV are common. Neitzel and Grimstad [68] reported that 91% of deer from three sites surrounding the greater Minneapolis-St. Paul, Minnesota metropolitan area were positive for JCV but they did not report on road densities within their study areas. In Nova Scotia, Canada 87.8% of the deer tested were positive for JCV [15].

Seropositive deer selected habitat that was further from rivers (moving water not conducive to mosquito breeding) and closer to anthropogenic areas. Containers in back yards provide mosquito breeding habitats when filled with stagnant water followed by warming temperatures [69–70]. Pecoraro et al [4] found that WNV mosquito vectors were most abundant in urbanized habitats in Seattle, Washington. Additionally, deer occupying urban landscapes frequent areas close to human dwellings particularly when anthropogenic foods (i.e., bird feeders) are available [64, 71]. This juxtaposition of host-vector-pathogen identifies a hotspot where outreach efforts could be used to educate residents about the risks of JCV and how to reduce vector habitat associated with urbanization [69–70]. Removing anthropogenic foods that concentrate host deer in urban environments and reducing densities of overabundant deer in the same communities is also warranted [71–72].

Although we presented evidence of correlations between JCV seropositive deer resource selection and various environmental conditions, we constructed this evaluation to understand population-level resource selection. We detected interaction effects between the environment and the spatial distribution of precipitation that require further investigation. We suggest that researchers focus their assessments at finer spatial and temporal resolutions to fully understand the environmental correlates for JCV. For example, understanding site specific environmental conditions with respect to mosquito abundance or infectivity would provide additional information for identifying local hotspots for JCV. Furthermore, with the advent of GPS tracking technology since this field study was conducted, one can validate predictive model output(s) using k-fold cross validation or bootstrapping techniques. This information is valuable for mapping the spatial configuration of hotspots for JCV and could be used to alter recreation use areas/habitats of humans and inform education initiatives effectively reducing the number of human exposures to the virus.
